# Investigating the relationship between public community landscape space and older adults’ health using structural equation modeling

**DOI:** 10.3389/fpubh.2025.1677759

**Published:** 2025-12-31

**Authors:** Chunjiao Chen, Dongjiao Chen, Shuang Wu

**Affiliations:** 1 AIGC Design Innovation Research Center, Faculty of Social Sciences, Beijing Institute of Technology, Zhuhai, Guangdong, China; 2 Guangzhou Liwan District Zhanqian Street Community Health Service Center, Guangzhou, Guangdong, China; 3 Research Institute of Beijing Institute of Technology, Zhuhai, Guangdong, China

**Keywords:** older adults’ health, community landscape space, physical activity, social participation, socioeconomic status (SES)

## Abstract

**Objective:**

This study examined how community landscape quality impacts older adults’ health through outdoor activity and social participation, while assessing socioeconomic status (SES) as a moderator, within China’s urbanization and aging context.

**Methods:**

A cross-sectional survey of 427 urban older adults measured landscape quality, physical activity, social participation, SES, and health (physical, mental, and satisfaction). SEM analyzed direct/indirect pathways and SES moderation.

**Results:**

Community landscape space quality had a significant direct effect on older adults’ health (*β* = 0.280, *p* < 0.001), as well as indirect effects through outdoor exercise (*β* = 0.120) and social participation (*β* = 0.083). The model explained 51.2% of the total variance in health outcomes. The moderating effect of SES was significant: low-SES groups derived stronger direct health benefits from environmental quality, whereas high-SES groups achieved health improvements more through behavioral pathways, revealing heterogeneity in the “environment-health” mechanism across different socioeconomic groups.

**Conclusion:**

The findings indicate that high-quality community landscape spaces effectively promote healthy aging among older adults by activating mechanisms such as physical activity and social participation. At the same time, pathway differences across SES groups suggest that future urban intervention strategies should account for social stratification characteristics to achieve health equity. This study uniquely integrates socio-ecological and environmental determinants frameworks to quantify multi-pathway influences on older adults’ health using SEM, underscoring both its originality and international relevance.

## 1 Introduction

A significant change in demographics during the 21st century, in addition to global health initiatives, is the rise of the world’s population over 60 years of age, which will nearly double by 2050 globally (1). This demographic transition will greatly affect demand on health systems and caregiving capacity. In this context, urban places are being recognized more as determinants of healthy aging (2). Specifically, the quality of community landscapes, such as the amount of green coverage, access, safety, aesthetics, and available amenities, has emerged as an essential building block of physical and psychological health and well-being in older adults. Studies show that communities with well-designed environments can motivate engagement with physical activity, decrease levels of loneliness, and increase social integration; activities that lessen the risk of chronic disease and declines in mental health (3–6).

While the spatial environment may impact health in several ways, such as access to or amount of green space, it may also involve safety and upkeep, and if we engage in prosocial activities (3, 4, 7). For example, spatial disparities such as low walkability and low green spaces in poorer parts of town/city could intensify health vulnerability (6, 8, 9). More generally, while green space may contribute to better health (10). Most studies do not investigate behaviors as mediators of health, such as physical activity and social engagement (11, 12), or report on inequalities based on socioeconomic status (SES) (13). More recent studies stress that environmental impacts on health are rarely direct effects, but rather mediated through behavioral pathways, such as physical activity or social engagement (8–10, 14). In addition, the inequitable distribution of resources means that these groups may have less favorable opportunities for behavior activation, thus making it an important modifier of the environment–health pathway (11–13, 15).

In this research project, we will formally define Public Community Landscape Space (PCLS) as the intersecting physical and perceived characteristics of outdoor community spaces—parks, walking paths, public squares, recreational settings, green space, safety features, aesthetics, and accessibility—that all interact to create the daily lived experience of older adults. By operationalizing PCLS, we will build on some of the narrower terms like “green space” and “built environment” to understand both objectively (e.g., availability of facilities or vegetation or lighting) and subjectively (e.g., safety, cleanliness, aesthetics) the multi-dimensionality of the environment as older adults perceive it in their communities (3, 7, 10).

The research is being situated in the social ecological theory (16), which allows for breadth of possible influence for health, and encourages a multi-level and data-driven analysis, with an environmental determinant of health (EDH) framework (17, 18) positioning spatial context as an active determinant of health. While historically the analyses of health, and the spatial context of the built environment have been framed in largely regression or multi-level modeling for methodological analyses (11, 12, 19) to try and refine statistics and understand learning about more nuanced relationships, it does not mean that behaviors or pathways can also be measured on the same plane with moderation, or yield a reflection of our learning about the latent constructs. Structural equation modeling (SEM), on the other hand, allows for the estimation of current, indirect, and moderated pathways manifested concurrently and presents a preferable methodology for understanding how environmental quality, mediated behaviors, and SES influence the health pathways.

Recent studies have started to link issues of landscape quality (community design) with the behavior pathways outcomes, namely, participatory governance (20, 21), sensory mediated restorative impacts (22), and attitudinal qualities toward uses (22). However, very little work has integrated SES stratification influences on pathways of behaviors. While the indicators used to measure the PCLS dimensions in this study (Table 1) were drawn from known frameworks on walkability, greenspace assessment, and age-friendly environments (23–31).

**Table 1:** Independent variable: quality of community landscape space.

Latent variable	Dimension	Key indicators	Measurement method	References (recent, supportive)
Quality of community landscape space	Accessibility	• Number of parks or plazas within 5-min walking distance • Completeness of barrier-free facilities • Perceived transportation convenience	On-site evaluation; resident surveys	(23–25)
	Greenness	• Percentage of green space coverage • Visual greenery index (vegetation visibility) • Perceived biodiversity of plant species	On-site measurement; resident perception survey	(26–28)
	Safety	• Adequacy of nighttime lighting • Spatial openness (absence of blind spots) • Incidence of safety-related events	Resident reports, community records, observer evaluation	(29, 30)
	Aesthetic Perception	• Aesthetic quality of landscape design • Harmony between architecture and green spaces • Cleanliness and maintenance frequency	Resident evaluation; researcher observation	(31)
	Facility Completeness	• Density of fitness equipment • Availability and quality of resting seats • Continuity of pedestrian walkways	On-site assessment; checklist scoring	(23)

While a robust body of evidence has established associations between the built environment and health outcomes in older populations (32, 33), several conceptual and methodological gaps necessitate further investigation. Existing research has demonstrated that neighborhood characteristics such as green space availability and walkability are closely linked to older adults’ physical and mental health (34, 35). However, most studies rely on objective environmental audits or single-dimensional indicators (e.g., land-use mix, intersection density), overlooking the multidimensional and subjective nature of residents’ environmental perceptions. A more holistic, resident-centered evaluation of public community landscape space (PCLS) quality—integrating perceptions of safety, aesthetics, amenities, and accessibility—remains relatively scarce in the literature (36). This gap limits our understanding of which aspects of the lived environment most strongly influence the wellbeing of older adults.

Furthermore, prior research often examines specific behavioral mediators such as outdoor physical activity or social participation in isolation, rather than within an integrated framework that captures their parallel mediating effects on health. Such separation hinders direct comparison of their relative influence and the potential synergistic mechanisms through which the built environment promotes active and healthy aging.

In addition, although socioeconomic inequalities in both health outcomes and environmental exposure are well documented (32, 34), the potential for SES to moderate these complex environment–behavior–health pathways remains underexplored. The beneficial impacts of high-quality community landscapes may not be equally distributed across social strata, highlighting the need to integrate an equity-oriented perspective into community design and aging research.

To address these interconnected gaps, this study makes a novel contribution by: (1) operationalizing a multi-dimensional PCLS quality construct that captures both objective features and subjective resident perceptions; (2) employing SEM to rigorously test the parallel mediating effects of outdoor exercise and social participation within an integrated framework; and (3) investigating the moderating role of SES on the proposed pathways. This integrated approach moves beyond examining isolated associations and offers a more nuanced understanding of the mechanisms through which community landscapes impact health, and for which subgroups these impacts are most pronounced. The findings aim to provide actionable insights for designing equitable community landscapes that effectively promote healthy aging for diverse older adult populations.

In conclusion, this study will bridge environmental, behavioral, and social elements into an SEM framework with multiple paths to provide evidence for mechanisms and discourse for further examining the possible policy implications for health equity issues with positive implications for older adult populations in urban spaces.

## 2 Methods

### 2.1 Study sample

A sample of 427 older individuals was obtained from four districts in Guangzhou, China (Liwan, Yuexiu, Haizhu, and Baiyun), classified as typical urban neighborhood communities with diverse landscape features. Communities selected for participation had to meet stratified sampling criteria to reflect community size, type of built environment, and socioeconomic diversity. Communities were eligible if at least 20% of the residents were aged 60 years or older and if they were a stable population (resided for ≥3 years). Recruitment of participants was conducted through neighborhood committees and community service organizations. The eligibility criteria for seeking individuals to take the survey were: (1) 60 years or older, (2) resided in the community for ≥1 year, and (3) independently able to complete the questionnaire or with minimal assistance. Individuals with severe cognitive impairment or major disabling conditions were screened out of the study.

Data collection included subjective survey responses and objective assessments of the landscape. Written informed consent was obtained from participants, and ethical approval was obtained from the Ethics Committee of South China University of Technology (Approval No. 2025SCUT0302).

The study design utilized a cross-sectional approach, and the data collection period was from February to May 2025 to facilitate appropriate seasonal conditions and minimize seasonal bias regarding environmental conditions. Data were collected by trained investigators, each with previous experience conducting field survey research.

The communities pieced together described different types of built environment — traditional dense neighborhoods, planned residential compounds, and newly developed community parks — in terms of spatial context, traditional commons landscape characteristics, and the presence of public “private” urban spaces.

**Figure 1:**
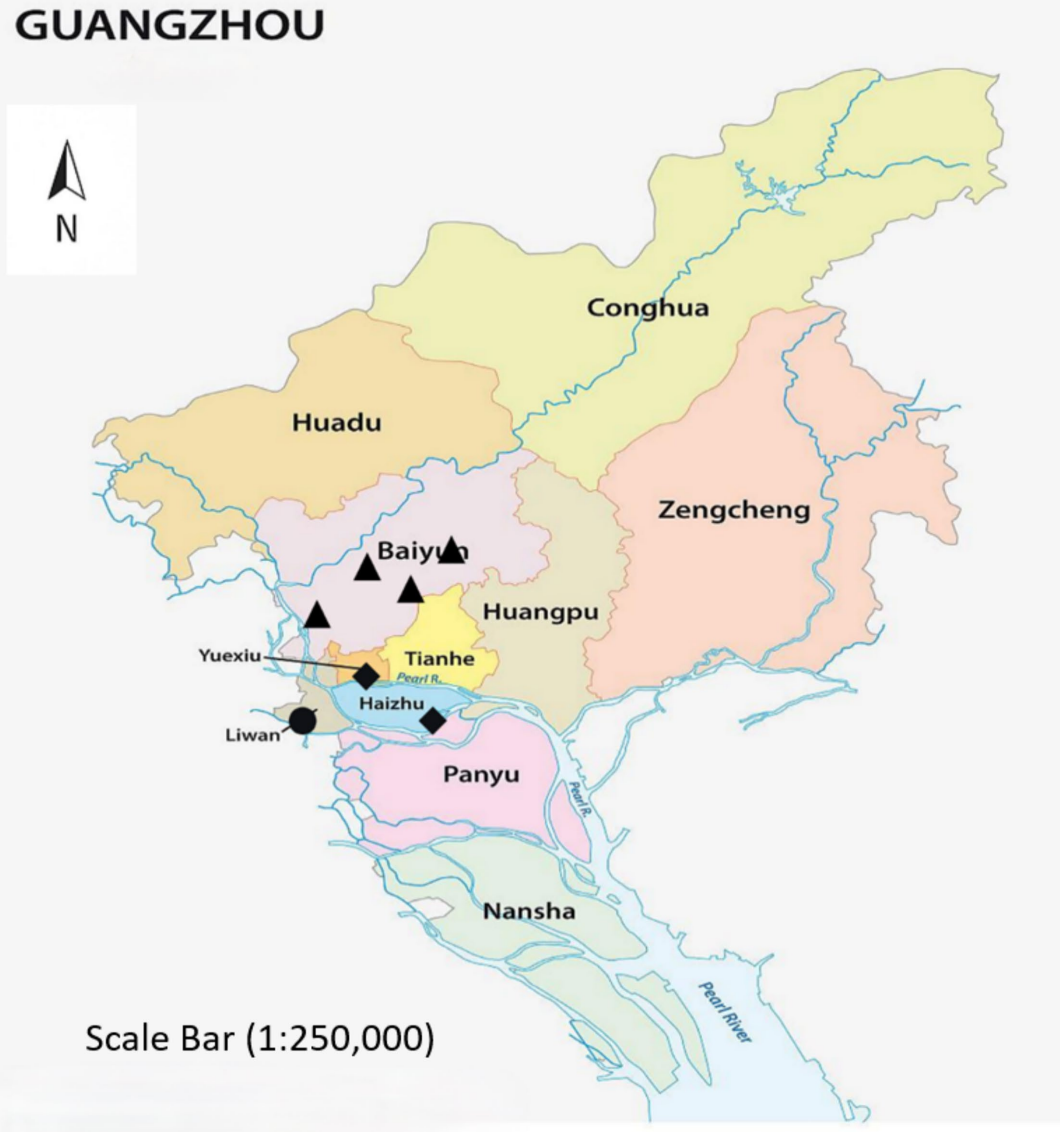
Study area map showing the distribution of sampled communities in Guangzhou, China. The three community landscape types are represented by different symbols: ● traditional dense neighborhoods, ◆ planned residential compounds, and ▲ newly developed community parks. Base-map data were obtained from the Guangzhou Municipal Planning Bureau (37) and were subsequently hand-drawn for illustrative purposes, and the coordinates of the sampled communities were recorded in the field using GPS from February to May 2025.

The spatial distribution of the surveyed communities is displayed in Figure 1 in the study area map of Guangzhou, China. The map indicates the urban districts (Liwan, Yuexiu, Haizhu, and Baiyun) and where each of the recruited communities meeting the inclusion criteria (>20% residents 60 years of age and older) was located. Data for the map base were sourced from the Guangzhou Municipal Planning Bureau (37) and the coordinates for the sampled communities were recorded with GPS in the field, collecting this data from February to May 2025.

Figure 1 includes the full base map of Guangzhou city, displaying the boundaries of the four urban districts (Liwan, Yuexiu, Haizhu, and Baiyun) and the specific locations of the sampled communities. A north arrow and scale bar are added to meet publication requirements and help readers identify the sampling sites within the city’s spatial context.

The sampled communities represent three major landscape feature types: (1) traditional dense neighborhoods with limited green space and narrow lanes (Liwan District, 108 samples); (2) planned residential compounds with medium-density greenery and open plazas (Yuexiu and Haizhu Districts, 184 samples); and (3) newly developed community parks with high greenery coverage and comprehensive facilities (Baiyun District, 135 samples). This classification ensures diversity in landscape types within the sampling framework.

### 2.2 Study design

#### 2.2.1 Selection of model indicators

Informed by socio-ecological theory (15) and the environmental determinants of health framework (18), we derived latent variables consistent with the framework with a “micro-spatial environment → behavioral patterns → health outcomes” logic. We identified indicators of the quality of landscape (accessibility, greenness, safety, aesthetics, and completeness of facilities) and health outcomes/behaviors from instruments validated in aging and urban-health contexts, to facilitate conceptual and measurement validity. Our indicators of landscape quality were developed from previously validated frameworks and empirical studies. Specifically, accessibility (i.e., walkability, barrier-free access, and proximity) aligned with efforts to study walkability and active aging (3, 4, 38); greenness (i.e., coverage, visual greenness, and perceived biodiversity) aligned with evidence-related vegetation restoration and activity (10, 39, 40); safety (i.e., adequate lighting, spatial openness) of the landscape builds upon the work conceptualizing safety and health-related outcomes, such as outdoor activity in older adults (4, 39). Aesthetics and cleanliness originated from studies exploring restorative environments, public space, and perceived quality (5, 7); and completeness of facilities (i.e., fitness and support equipment, resting space, walkways) was from the age-friendly city and public health planning frameworks (3, 4, 40). This approach was intended to allow mean values to be estimated by studies previously conducted while reflecting the multidimensionality of the public community landscape.

#### 2.2.2 Dependent variable: health of older adults

Health status was conceptualized as three related, but distinct dimensions (Table 2) (41–46). Physical health included the number of chronic diseases, Activities of Daily Living (ADL), and mobility limitation. Mental health was assessed with the Geriatric Depression Scale–Short Form (GDS-SF), UCLA Loneliness Scale (short form), and Generalized Anxiety Disorder-2 (GAD-2) in the brief measures commonly used in gerontological research (47, 48). Health satisfaction was a single five-point self-rated item.

**Table 2:** Dependent variable: health of older adults.

Latent variable	Dimension	Observed indicators	Measurement method	References (recent, supportive)
Health of older adults	Physical health	Number of chronic diseases; activities of daily living (ADL) score; limitation in mobility	Self-reported survey items, standard ADL scale, functional mobility tests	(41, 42)
	Mental health	Geriatric Depression Scale-Short Form (GDS-SF); UCLA Loneliness Scale (Short Form); Generalized Anxiety Disorder-2 (GAD-2)	Self-administered psychological assessments; structured surveys	(43–45)
	Health satisfaction	Overall self-rated health satisfaction (single-item Likert 5-point scale)	Self-reported	(46)

#### 2.2.3 Independent variable: quality of community landscape space

The quality of community landscape space was conceptualized as the overall attributes of community open spaces based on older adults’ subjective perspective and objective experiences, and its measurement included five dimensions, which were each based on multiple observed indicators (Table 2) (41–46). Each indicator was rated on a 5-point Likert scale (1 = very poor, 5 = very good). Exploratory and confirmatory factor analyses (EFA and CFA) were conducted to support the dimensionality and stability of the latent variable structure.

Indicator rationale and precedent: Accessibility (walkability, barrier-free access, proximity) and greenness (coverage, visual greenery) were considered core determinants of physical activity and restoration (10, 38, 40); Safety (lighting, openness) is supportive of out-of-home activity among older adults (4, 39). Aesthetics and cleanliness are linked to perceived quality/restorative Ness (5, 7); Facility completeness (fitness equipment, benches, connectivity of walkways) supports use by older adults (3, 38, 40).

#### 2.2.4 Mediating variables: outdoor exercise and social participation

Outdoor exercise was operationalized using two indicators: the weekly frequency of outdoor physical activities (e.g., walking and jogging) and the average duration per session, both measured via self-reported questionnaires. Social participation was assessed using a multidimensional approach, including the frequency of participation in five types of activities over the past year: volunteer work, mutual aid groups, interest clubs, community governance meetings, and cultural activities. The frequency of each activity was measured on a 5-point Likert scale (ranging from 1 = never to 5 = twice or more per week). These two mediating variables were expected to partially explain the relationship between community landscape space quality and older adults’ health outcomes.

The measurement indicators for both mediating variables were adapted from established research rather than being developed solely by the authors. Specifically, the operationalization of social participation and outdoor activity referred to prior gerontological studies that validated comparable constructs of community engagement and physical activity among older adults (49, 50). Minor contextual adjustments were made to fit the Chinese urban community context.

#### 2.2.5 Moderating variable: socioeconomic status (SES)

SES was determined as a composite score (*z*-score) with standardized measures from education level, personal monthly income, and prior occupational category, and then divided into high and low groups based on the median. Previous occupation was categorized into five occupational categories (1 = elementary/manual; 2 = services/sales; 3 = clerical/skilled craft; 4 = professional/technician; 5 = managerial/administrator), and standardized as well, before being a part of the composite score. Sensitivity checks with alternative two- and three-level occupational groups produced qualitatively similar results.

### 2.3 Control variables

To minimize confounding, models adjusted for gender (0 = male, 1 = female), age (years), education (total years of formal schooling), and length of residence in the current community (years).

### 2.4 Model construction

We developed a SEM that is multi-path, multi-dimensional, in which landscape quality (a second-order latent construct and five first-order dimensions) directly affects an older adult’s health (three dimensions that define health: physical health, mental health, and health satisfaction) through two parallel mediating paths (outdoor exercise and social participation). The model also included a direct path from landscape quality to health. Sociodemographic status was included in the model as a moderator to assess potential differences in the key paths. We used maximum likelihood estimation for its positive asymptotic properties demonstrated in complex modeling. All path coefficients were reported as standardized *β* for ease of comparison among the pathways. The modeling framework followed a causal-process reasoning from input (landscape quality) to process (behavioral mediation) to outcome (health outcomes) is consistent with socio-ecological and health behavior theory; instead of multiple regression, we opted for SEM because it can simultaneously estimate latent constructs, multiple mediations, moderation, and formally decompose effects (11, 12, 19).

### 2.5 Research hypotheses

Guided by the socio-ecological model (16) and the Environmental Determinants of Health (EDH) framework (17, 18), this study formulates five hypotheses to explain how Public Community Landscape Space (PCLS) influences the health of older adults through both behavioral and socioeconomic pathways. Each hypothesis is grounded in theoretical reasoning and supported by empirical findings from prior studies.

*H1* – Direct effect of landscape quality on health.

High-quality PCLS exerts a direct positive effect on the physical, mental, and subjective health of older adults. Prior research shows that access to green, safe, and aesthetically pleasing environments offers restorative experiences, mitigates stress, and enhances overall well-being (5, 22).

*H2* – Mediating role of outdoor exercise.

Well-designed landscape environments encourage outdoor physical activity by improving walkability, accessibility, and perceived safety. Regular outdoor exercise strengthens cardiovascular and musculoskeletal function and enhances vitality, thereby serving as a behavioral pathway linking landscape quality to health outcomes (3, 4, 20).

*H3* – Mediating role of social participation.

Pleasant and inclusive landscape spaces foster social participation, offering opportunities for interaction, friendship, and civic engagement. Higher levels of social participation alleviate loneliness, enhance emotional well-being, and consequently mediate the relationship between landscape quality and health (7).

*H4* – Parallel mediation of outdoor exercise and social participation.

Outdoor exercise and social participation function as parallel and complementary mediators through which landscape quality influences health. This aligns with socio-ecological models emphasizing that multiple behavioral pathways operate simultaneously to promote healthy aging (11, 12, 19).

*H5a* – Moderating effect of socioeconomic status (SES).

Socioeconomic status is expected to moderate the strength of the association between landscape quality and health. Older adults with lower SES may derive stronger direct health benefits from improvements in public landscape quality, as they have fewer private resources for recreation and wellness (40).

H5b – Differential behavioral effects among high-SES groups.

Conversely, older adults with higher SES are more likely to demonstrate indirect benefits through behavioral engagement—such as increased outdoor activity and social interaction—when high-quality landscapes are available (14, 19).

Together, these five hypotheses establish a comprehensive conceptual framework linking environmental quality and behavioral engagement within the socio-economic context of urban aging. The model highlights how differences in environmental exposure and individual resources may jointly shape health disparities among older adults living in diverse community settings.

### 2.6 Data collection methodology

The research employed a rigorously designed cross-sectional study framework, implemented between February and May/2025, to control for seasonal variations. A team of trained field researchers conducted comprehensive data collection through: (1) standardized face-to-face interviews using validated questionnaires administered at designated community locations (service centers, activity hubs, and public spaces); (2) systematic environmental assessments where two independent evaluators objectively quantified landscape characteristics using predefined measurement protocols. The final analytical sample comprised 427 eligible older adult participants who provided complete responses across all key variables. Quality assurance involved double-entry verification and consistency checks; interviewers used a common script, 10% of interviews were double-checked by phone, and a 10% subsample of environmental audits was scored in common to examine inter-rater agreement (target ≥ 0.80). Primary outcome variables were complete-case; for auxiliary variables with little missingness (<5%), we used multiple imputation prior to CFA/SEM to retain power and account for missingness, as explained below.

### 2.7 Analytical procedures

The analyses utilized SPSS 26.0 and AMOS 26.0. Data preparation began with the elimination of cases exceeding 15% missingness on critical variables for the purposes of ensuring complete-case analyses of the primary constructs. Any remaining cases with item-level missingness below 5% received attention through multiple imputation (five datasets) prior to CFA/SEM, noting that only minimal gaps were imputed for auxiliary items while core variables remained complete. Univariate diagnostics assessed distributions and potential outliers, while the measure of validation through confirmatory factor analysis considered psychometric properties (target loadings ≥ 0.60, AVE > 0.50, CR > 0.70), as well as discriminant validity through the Fornell–Larcker criterion and goodness of fit models as determined by *χ*^2^/df < 3.0, CFI/TLI > 0.90, and RMSEA/SRMR < 0.08. In structural modeling, maximum likelihood estimation was used to assess the hypothesized pathways with standardized coefficients, and the model was adjusted as theory warranted, utilizing modification indices. Mediation was analyzed with bias-corrected bootstrapping with 5,000 samples; indirect effects are significant if the 95% confidence intervals include zero, and effect decompositions provide a ratio of indirect effects’ contributions to a given pathway. Group comparisons for moderation and invariance testing were accomplished by comparing groups with high SES against low SES after addressing configural/metric invariance. Specifically, chi-square difference tests (Δ*χ*^2^) were utilized to compare the path estimates between groups to determine significance at *p* < 0.05.

## 3 Results

### 3.1 Descriptive statistics

The study sample consisted of older adults with a mean age of 69.3 years (SD = 6.8), of whom 55% were female. Participants had an average of 8.6 years of formal education, with significant differences observed between socioeconomic groups (high-SES: 9.2 years; low-SES: 7.9 years). In terms of health outcomes, participants reported an average of 1.47 chronic conditions (SD = 1.06), with the high-SES group demonstrating better physical health indicators, including fewer chronic diseases (1.26 vs. 1.68), better functional ability, and fewer mobility limitations compared to their low-SES counterparts. Mental health assessments revealed that high-SES participants scored significantly lower on measures of depression, loneliness, and anxiety, while also reporting higher levels of health satisfaction (3.80 vs. 3.38).

Community landscape quality assessments across five dimensions (accessibility, greenery, safety, aesthetics, and facility completeness) yielded moderately positive scores (range: 3.35–3.83). Significant socioeconomic disparities were observed in environmental perceptions, with high-SES participants consistently rating their community environments more favorably, particularly in areas of green space coverage, cleanliness, and accessibility features. Behavioral data showed participants engaged in outdoor exercise an average of 4.20 times per week for approximately 28.6 min per session, with minimal socioeconomic variation. However, social participation patterns differed markedly, with high-SES individuals demonstrating significantly greater involvement in community activities and organizations (Table 3).

**Table 3:** Descriptive statistics of observed variables by SES group (*n* = 427).

Variable category	Observed variable	Description	Total (*M* ± SD)	Low SES (*M* ± SD)	High SES (*M* ± SD)
Health of older adults	PH1	Number of chronic diseases	1.47 ± 1.06	1.68 ± 1.11	1.26 ± 0.97
	PH2	ADL (activities of daily living) score	87.4 ± 10.5	84.6 ± 11.8	90.3 ± 9.0
	PH3	Mobility limitation (reverse-coded)	3.42 ± 0.78	3.21 ± 0.81	3.63 ± 0.72
	MH1	GDS-SF (depression) score	3.15 ± 2.31	3.52 ± 2.50	2.74 ± 2.01
	MH2	Subjective loneliness (UCLA short form)	2.83 ± 0.91	3.02 ± 0.88	2.62 ± 0.93
	MH3	GAD-2 (anxiety) score	1.67 ± 1.22	1.89 ± 1.30	1.42 ± 1.09
	HS1	Self-rated health satisfaction	3.61 ± 0.96	3.38 ± 0.93	3.80 ± 0.95
Quality of landscape space	ACC1	Parks within a 5-min walk	3.48 ± 1.05	3.21 ± 1.08	3.71 ± 1.01
	ACC2	Barrier-free access completeness	3.35 ± 0.92	3.08 ± 0.94	3.64 ± 0.89
	ACC3	Perceived transportation convenience	3.62 ± 0.87	3.35 ± 0.88	3.86 ± 0.82
	GRE1	Green space coverage	3.75 ± 0.90	3.44 ± 0.91	4.02 ± 0.80
	GRE2	Visibility of vegetation	3.83 ± 0.85	3.58 ± 0.83	4.04 ± 0.78
	GRE3	Perceived biodiversity	3.57 ± 0.95	3.31 ± 0.97	3.78 ± 0.88
	SAF1	Nighttime lighting adequacy	3.70 ± 0.91	3.43 ± 0.96	3.94 ± 0.85
	SAF2	Spatial openness (few blind spots)	3.48 ± 0.88	3.22 ± 0.85	3.74 ± 0.83
	SAF3	Safety event frequency (reverse-coded)	3.85 ± 0.82	3.59 ± 0.90	4.06 ± 0.76
	AES1	Aesthetic quality of design	3.62 ± 0.89	3.28 ± 0.91	3.93 ± 0.80
	AES2	Spatial harmony (architecture-nature)	3.55 ± 0.92	3.21 ± 0.94	3.85 ± 0.85
	AES3	Cleanliness and maintenance	3.80 ± 0.87	3.42 ± 0.90	4.12 ± 0.76
	FAC1	Fitness equipment availability	3.36 ± 0.88	3.09 ± 0.92	3.63 ± 0.78
	FAC2	Resting seat sufficiency	3.52 ± 0.90	S3.25 ± 0.88	3.78 ± 0.86
	FAC3	Walkway continuity and maintenance	3.67 ± 0.91	3.33 ± 0.93	3.94 ± 0.82
Mediators	OE1	Outdoor exercise frequency (times/week)	4.20 ± 2.90	4.08 ± 2.87	4.26 ± 2.87
	OE2	Average duration of exercise (min/session)	28.6 ± 33.6	28.3 ± 26.5	26.5 ± 42.0
	SP1	Participation in social activities	2.71 ± 0.91	2.58 ± 0.88	2.85 ± 0.92
	SP2	Diversity of social participation	2.63 ± 0.85	2.52 ± 0.83	2.74 ± 0.88
Control variables	Age	Age (years)	69.3 ± 6.8	70.2 ± 6.5	68.4 ± 7.2
	Gender	0 = Male, 1 = Female	0.55 ± 0.50	0.56 ± 0.49	0.53 ± 0.50
	Education	Years of formal education	8.6 ± 3.1	7.9 ± 3.0	9.2 ± 3.0
	Length of residence	Years living in current community	17.2 ± 9.4	18.1 ± 10.1	16.5 ± 8.7

### 3.2 Measurement model validation

Before assessing the full structural model, we specifically conducted a preliminary confirmatory factor analysis (CFA) of all latent (or unobserved) variables (i.e., the measurement model). Results indicated that standardized factor loadings were within the range of 0.68 and 0.92 (all *p*s < 0.001), suggesting that the observed variables satisfactorily captured the intended constructs. The fit indices also showed acceptable fit (*χ*^2^/df = 2.41, CFI = 0.958, TLI = 0.946, RMSEA = 0.058, SRMR = 0.045). Together, these results provided considerable evidence of the goodness of fit of the measurement structure. Convergent validity was demonstrated [all constructs had Average Variance Extracted (AVE) greater than 0.50 and Composite Reliability (CR) greater than 0.70]. Cronbach’s *α* coefficients also showed internal consistency, where most alphas exceeded 0.70; and discriminant validity was supported through the Fornell–Larcker criterion through the squared roots of the AVE being greater than the inter-construct correlations. Together, these preliminary CFA results provide evidence that the measurement model has acceptable psychometric properties and is appropriate to advance in the subsequent SEM (refer to Table 4: Preliminary CFA).

**Table 4:** Construct reliability and validity of latent variables.

Main constructs	Items	Outer loadings	Cronbach’s *α*	CR	AVE
Physical health	PH1: Number of chronic diseases	0.692	0.756	0.826	0.613
	PH2: ADL score	0.843			
	PH3: Mobility limitation	0.801			
Mental health	MH1: Depression (GDS-SF)	0.779	0.723	0.809	0.588
	MH2: Loneliness (UCLA)	0.804			
	MH3: Anxiety (GAD-2)	0.713			
Health satisfaction	HS1: Self-rated health satisfaction	0.863	0.795	0.863	0.756
Accessibility	ACC1: Parks within 5-min walk	0.701	0.771	0.839	0.566
	ACC2: Barrier-free access	0.79			
	ACC3: Transport convenience	0.798			
Greenness	GRE1: Green space coverage	0.747	0.768	0.832	0.553
	GRE2: Visible vegetation	0.732			
	GRE3: Biodiversity perception	0.742			
Safety	SAF1: Lighting adequacy	0.782	0.74	0.817	0.537
	SAF2: Spatial openness	0.72			
	SAF3: Crime rate perception (reverse)	0.683			
Aesthetic perception	AES1: Landscape aesthetics	0.803	0.789	0.857	0.669
	AES2: Harmony of built-natural	0.801			
	AES3: Cleanliness	0.824			
Facility completeness	FAC1: Fitness equipment	0.738	0.766	0.83	0.62
	FAC2: Resting seats	0.804			
	FAC3: Walkway continuity	0.799			
Outdoor exercise	OE1: Frequency of walking	0.768	0.726	0.829	0.708
	OE2: Walking time per session	0.921			
Social participation	SP1: Participation frequency	0.765	0.782	0.846	0.581
	SP2: Participation diversity	0.83			

### 3.3 Structural model testing

The structural model exhibited a good fit (*χ*^2^/df = 2.37, CFI = 0.961, TLI = 0.950, RMSEA = 0.057, SRMR = 0.041) to the data. As per the reviewer’s suggestions, Table 5 has been modified to have a single combined *R*^2^ row to reduce confusion. The model indicated that community landscape quality had a direct and significant positive impact on older adults’ health (*β* = 0.280, *t* = 5.34, *p* < 0.001) and indirect effects of landscape quality on older adults’ health through outdoor exercise (*β* = 0.467, *t* = 9.12, *p* < 0.001) and social participation (*β* = 0.423, *t* = 8.01, *p* < 0.001); both mediating variables also significantly predicted health outcomes. Outdoor exercise (*β* = 0.256, *t* = 4.88, *p* < 0.001) and social participation (*β* = 0.197, *t* = 3.92, *p* < 0.001) both positively predicted health in a manner that is independent of each other.

**Table 5:** Structural model path coefficients, significance, and *R*^2^ values.

Path	*β*	*t*	*p*	Significant
Landscape quality → Health	0.28	5.34	<0.001	Yes
Landscape quality → Outdoor exercise	0.467	9.12	<0.001	Yes
Landscape quality → Social participation	0.423	8.01	<0.001	Yes
Outdoor exercise → Health	0.256	4.88	<0.001	Yes
Social participation → Health	0.197	3.92	<0.001	Yes
*R*^2^ (Health)	0.512	—	—	
*R*^2^ (Outdoor exercise)	0.332	—	—	
*R*^2^ (Social participation)	0.298	—	—	

**Figure 2:**
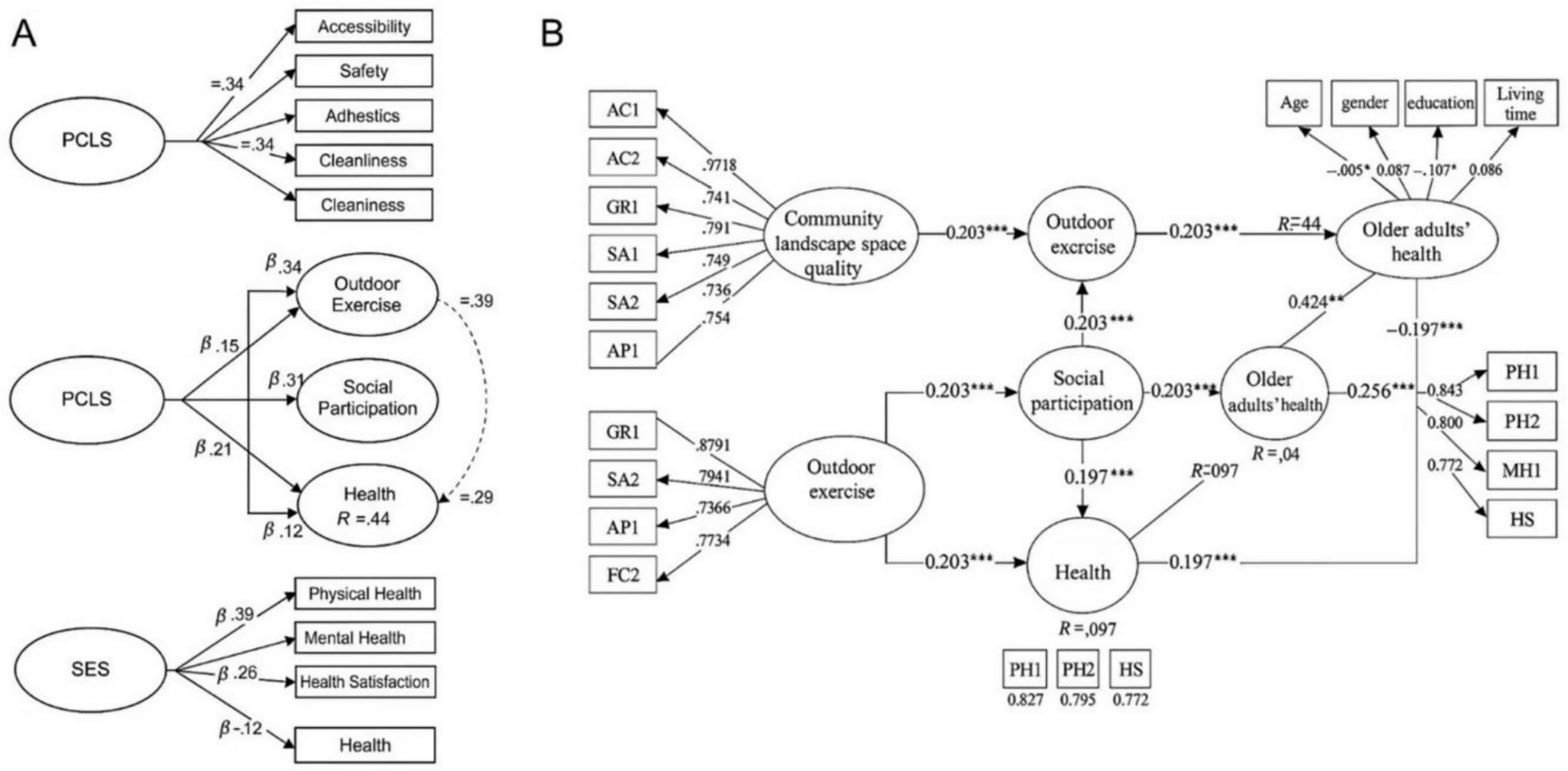
Structural equation model showing standardized path coefficients and explained variance (*R*^2^) for dependent variables. **(A)** The direct pathways from landscape quality to health; **(B)** the parallel mediators (i.e., outdoor exercise, social participation) to visualize the total impact. ***p < 0.001, **p < 0.01, *p < 0.05.

To enhance clarity and follow the recommendations of the reviewer, results have now been visualized in two SEM diagrams, whereby Figure 2A depicts the direct pathways from landscape quality to health, and Figure 2B represents the parallel mediators (i.e., outdoor exercise, social participation) to visualize the total impact. We also wrote out “Social participate” as “Social participation” instead of beginning with the word “Social” and corrected duplicated latent variables accordingly.

### 3.4 Multi-group analysis by SES

To test the mediating paths in hypothesis three, we conducted bias-corrected bootstrapping with 5,000 resamples. Table 6 shows that the indirect effect through outdoor exercise (0.120, *t* = 4.36, *p* < 0.001; 95% CI [0.072, 0.178]) and social participation (0.083, *t* = 3.44, *p* = 0.001; 95% CI [0.038, 0.142]) were both above zero. Both significance tests indicated that there were statistically significant mediation effects since the confidence intervals did not include zero. These results suggest partial mediation, as the direct effect related to reporting health’s wellbeing (*β* = 0.280, *p* < 0.001) was also significant. Considering both the outdoor exercise and social participation, the total indirect effect is 0.203 (*t* = 5.91, *p* < 0.001; 95% CI [0.142, 0.270]). Together, these findings demonstrate strong evidence that there exist behavioral mechanisms that mediate the relationship between neighborhood design, age-friendly cities, or community landscape quality, and health/wellbeing. Through the mediators, neighborhood design or community landscape quality has indirect effects on health/wellbeing, but the direct path remains significant. As shown in Figure 2B, the health latent variable, *R*^2^ = 0.512, has a substantive explanatory power (see Table 7).

**Table 6:** Bootstrap mediation effect results (5,000 resamples).

Pathway	Indirect Effect	SE	*t*	*p*	95% CI	Significant
Landscape → Outdoor exercise → Health	0.12	0.028	4.36	<0.001	[0.072, 0.178]	Yes
Landscape → Social participation → Health	0.083	0.024	3.44	0.001	[0.038, 0.142]	Yes
Total Indirect Effect	0.203	0.034	5.91	<0.001	[0.142, 0.270]	Yes

**Table 7:** Total, direct, and indirect effects of the overall model path.

Independent variable	Intermediate variable	Dependent variable	Total effect	Direct effect	Indirect effect
Landscape space quality	Social participation	Older adults’ health	0.203***	0.280***	0.120***
Landscape space quality	Outdoor exercise	Older adults’ health	0.203***	0.280***	0.083***
Social participation	–	Older adults’ health	0.197***	0.197***	–
Outdoor exercise	–	Older adults’ health	0.256***	0.256***	–

***p < 0.001.

To test moderation by socioeconomic status, we used a multi-group SEM to compare high- and low-SES. Both models showed good fit (high SES: *χ*^2^/df = 2.19, CFI = 0.958, RMSEA = 0.055; low SES: *χ*^2^/df = 2.35, CFI = 0.952, RMSEA = 0.058), indicating evidence of configural invariance. We also conducted a chi-square difference test that compared the constrained and unconstrained models. This test indicated a significant difference in cross-group paths (Δ*χ*^2^ = 14.82, df = 3, *p* = 0.002). The direct effect of landscape quality on health was stronger for the low SES group (*β* = 0.31, *p* < 0.001) compared to the high SES group (*β* = 0.11, *p* = 0.088), suggesting a greater sensitivity to the environment for low SES populations as compared to high SES populations. Meanwhile, the high SES group had stronger behaviorally-mediated pathways, including landscape quality → outdoor exercise (*β* = 0.52, *p* < 0.001), and landscape quality → social participation (*β* = 0.46, *p* < 0.001). These pathways had downstream effects on health (outdoor exercise → health: *β* = 0.28, *p* < 0.01; social participation → health: *β* = 0.24, *p* < 0.01). The low SES group had mediated effects, but they were weaker (outdoor exercise → health: *β* = 0.19, *p* < 0.05; social participation → health: *β* = 0.17, p < 0.05). This analysis shows SES-related heterogeneity within the environment–health mechanism (Table 8).

**Table 8:** Multi-group SEM path coefficients by SES group.

Path	Low SES (*β*)	High SES (β)	Δ*β*	Δ*χ*^2^	*p*
Landscape quality → Health	0.31***	0.11	0.2	8.17	0.004
Landscape quality → Outdoor exercise	0.38***	0.52***	−0.14	5.02	0.025
Landscape quality → Social participation	0.29***	0.46***	−0.17	6.73	0.009
Outdoor exercise → Health	0.19*	0.28***	−0.09	3.95	0.047
Social participation → Health	0.17*	0.24**	−0.07	3.6	0.058

***p < 0.001, **p < 0.01, *p < 0.05.

## 4 Discussion

Community contexts do not solely function as edifices, but also function as agents that influence older adults’ physical activity, social activity, and psychological restoration (22, 38, 39, 47, 48, 51). In support of contemporary “healthy aging” frameworks that acknowledge determinants of the built environment, healthcare, and individual behaviors, we provide evidence that landscape quality is an important social determinant of older adults’ health.

The pathways provided in the structural equation model illustrate two critical mediations. Age-friendly outdoor spaces increased the prevalence of physical activity for older adults. This finding corroborates much of the existing evidence that environmental safety, easy walkability, and beauty influence behavior change (52, 53). Likewise, landscape quality facilitated social interactions – especially informal interactions, contributing to measurable improvements to mental health through increases in social support and declining feelings of loneliness – potentially consistent with Chen and Feeley’s (7) STIP Model of social capital as a protective factor. Though other studies have identified perceived restrictiveness of parks in Taipei (27, 28), and their connections to psychological wellbeing for older adults, this study reinforces that idea and provides some evidence that landscape quality may have a direct effect on health outcomes for older adults even when taking into account behavioral measures. Landscape quality certainly indicates other possible psychological pathways, demonstrating that landscape quality is multidimensional and is associated with the health of older adults in more complex ways.

The SES in this study served as a key moderator. People of lower SES demonstrated a stronger direct health benefit from the environmental improvements. This aligns with compensation theory (49, 50), where the quality of the landscape is a significant health asset for marginalized communities. In contrast, older adults of higher SES indicated a stronger behavioral mediation effect, which may be a result of having access to other private resources in town (i.e., gym, senior club, wellness programs). This two-pronged result confirms the value of equity in landscape interventions; improving the quality of public landscapes leads to outsize benefits for marginalized communities, followed by overall population benefits from behavioral activation programs (e.g., exercise programs, social events).

The unique aspect of this research was incorporating socio-ecological theory, environmental determinants of health, and restorative environment research in an explanatory multilevel model. More specifically, the research incorporated structural equation modeling (SEM) to concurrently test for mediation and moderation, which provides a potentially holistic view of how environmental quality, behavioral mediation, and SES all intersect. This is a step forward from research that typically applies regression models. Additionally, the moderating effect of SES provides emerging evidence of stratified health effects; environmental interventions may differentially impact health across SES.

There are implications of this research for high-density Asian cities and low-density Western cities globally. For example, small neighborhood spaces in Asian cities can be considered health resources for older adults, while parks in larger places in Western cities may serve in this capacity differently. Comparative studies could test the effect of landscape quality serving as a compensation for health outcomes for less favorable populations across cultural and urban locations, and provide recommendations for international urban planning.

While the findings of this study offer valuable implications for both high-density Asian cities and lower-density Western contexts, their applicability should be interpreted with caution. The extent to which community landscape characteristics influence residents’ health may vary with differences in urban form, social structure, and public space governance.

For example, studies in Western low-density cities, such as those in Australia and northern Europe, have shown that neighborhood accessibility, walkability, and social cohesion are stronger determinants of older adults’ physical activity and wellbeing than population density itself (54, 55). Conversely, in compact Asian megacities such as Tokyo and Hong Kong, research indicates that the quality and greenery of micro-scale public open spaces—rather than urban density—play a more critical role in shaping older adults’ health and social interaction outcomes (56, 57). Moreover, in lower-density Western contexts, automobile dependency, the role of private gardens, and different social norms surrounding public space use may further influence these relationships, making local park accessibility potentially even more crucial where destinations are farther apart.

Therefore, the generalizability of the present findings lies not in replicating specific spatial configurations but in emphasizing resident-centered landscape design and equitable access to restorative public spaces as universal principles for promoting active and healthy aging across diverse urban settings. Rooted in the context of a high-density Chinese city, this study contributes a validated methodological framework and a set of testable hypotheses for future comparative research. Applying this model to varied urban contexts will help clarify which mechanisms—such as outdoor exercise and social participation—represent universal pathways, and which are context-dependent, thereby advancing cross-cultural understanding in environmental gerontology.

From a practical point of view, walking or cycling improvements for neighborhoods are viewed as public health investments that will maximize returns in the disadvantaged neighborhoods. Like studies with Chang et al. (58) restorative perceptions, or Zhang et al. (59) when determining the activity mediated pathways, show urban glory is more appropriate in low-SES than high-SES. Universal design approaches will emphasize walkability, safety, and cleanliness across age and SES populations. Adding in community programs can develop improvement points with the physical changes that will generate virtuous “landscape-behavior-health” links together to enhance both physical and social health, and all community members.

There are a number of caveats to keep in consideration. First, because of the cross-sectional design of the study, the researcher also cannot assume causality; longitudinal or quasi-experimental studies studying paths would do this. Second, this research was based on measurement, and this has better potential for bias; Arthur C. Inkles’ study of GPS tracking, biometrics, and environmental data collected around geospatial information systems (GIS) would improve objectivity. Third, the SES index may serve better as three measures of socio-economic condition, with respect to income, education, and occupation, respectively. Fourth, the exploration in the study may have more implications and limitations because it used a single urban context. More work across multiple cities might improve external validity. Finally, it did not model or test a number of community types or economic conditions, which ultimately led to the reported underrepresentation of the complexity of the heterogeneity of the contexts.

For future studies, a research agenda that would more so in this order be – (a) pulls in the frame, looking for both path with and without model structure, (b) pulls in multiple community types, and (c) explores integrative approaches using digital technologies such as health app or wearables together with approaches to enhance the physical components in urban aging health measures.

## 5 Conclusion

This study found evidence that community landscape quality is a significant predictor of health in older adults; it influences health in older adults through physical activity and social engagement, and it operates through socioeconomic status. Given the methodology of a structural equation model, this study highlights the inclusion of socio-ecological theory and environmental determinants of health to create an alternative and novel multi-path framework for health within the environmental context for older adults.

The implications of this study yield firm policy recommendations, as advances in community-specific greenery, accessibility, and aesthetics yield important health benefits, especially in marginalized communities. These advances could be coupled with considerable modifications to community features, particularly those that prompt the residents to have behaviors that promote healthy aging. Perhaps most importantly, the results and findings possess significance in contexts outside of North America; if generalizable, the findings apply across densely urbanized contexts in Asia, and on the opposite end of the urban design spectrum, lower-density urbanism settings in the western world, bringing together multiple “environment-behavior-health” paradigms worldwide.

## Data Availability

The raw data supporting the conclusions of this article will be made available by the authors, without undue reservation.
